# Characterising concurrent pain experience and dietary patterns in people with chronic musculoskeletal pain: a feasibility study protocol

**DOI:** 10.1186/s40814-023-01438-4

**Published:** 2024-01-22

**Authors:** Susan J. Ward, Alison M. Coates, Katherine L. Baldock, Carolyn Berryman, Alison M. Hill

**Affiliations:** 1 Alliance for Research in Exercise, Nutrition and Activity (ARENA), University of South Australia, GPO Box 2471, Adelaide, South Australia 5001 Australia; 2 Clinical and Health Sciences, University of South Australia, GPO Box 2471, Adelaide, South Australia 5001 Australia; 3 Allied Health and Human Performance, University of South Australia, Adelaide, Australia; 4 Innovation IMPlementation And Clinical Translation (IIMPACT), University of South Australia, Adelaide, Australia

**Keywords:** Diet, Diet quality, Lifestyle, Pain, Musculoskeletal pain, Feasibility, Protocol

## Abstract

**Introduction:**

Nutrition-related factors linked to pain chronicity and disability include weight status and dietary behaviours. Dietary patterns associated with concurrent pain episodes, however, remain poorly characterised. This paper outlines the protocol for a feasibility study that aims to characterise pain-related dietary and lifestyle behaviours in people experiencing chronic musculoskeletal pain.

**Methods:**

The study will recruit participants who experience musculoskeletal pain on 5 or more days of the week for at least 3 months. Participants will attend two in-person clinic visits where physical measurements and a series of pain and lifestyle questionnaires will be completed. Visits will be conducted pre and post a 2-week self-monitoring period where participants will self-report concurrent diet, sleep, mood, and pain on four days and will wear a wrist-worn activity monitor (GENEActiv). Key feasibility metrics will evaluate participant recruitment, enrolment and retention rates, and compliance with the study data collection protocol.

**Discussion:**

There remains a lack of evidence behind dietary advice as an adjunct pain management tool. Upon completion of the protocol, feasibility outcomes will identify challenges to guide the design and delivery of a dietary intervention for chronic musculoskeletal pain.

**Supplementary Information:**

The online version contains supplementary material available at 10.1186/s40814-023-01438-4.

## Background

Musculoskeletal disorders are the largest contributor to years lived with a disability (YLDs) worldwide. Globally, musculoskeletal conditions account for 17% of all YLDs, with low back pain the main contributor [1]. Musculoskeletal conditions are characterised by pain (often chronic), and limitations to functioning, impacting a person’s ability to work, socialise, and engage in physical activity [2]. Evidence-based guidelines for the non-invasive management of chronic musculoskeletal pain (CMP) include therapeutic exercises, education, and advice to stay physically active [3, 4]. Current approaches to reduce the individual and economic burden of CMP acknowledge the complex multi-faceted nature of CMP and address modifiable lifestyle factors, such as physical activity and sedentary behaviours, sleep, mood, and dietary behaviours [3, 5].

Among lifestyle factors, dietary behaviours, independent of weight status, influence the prevalence, experience, and maintenance of persistent pain in an individual [6]. Dietary factors positively associated with the presence of chronic pain include higher intake of protein, fat, added sugar, and excess total energy intake [7]. Additionally, individuals with chronic pain have reduced intake of whole grains and fibre, and consume less fruit [8]. This dietary pattern is consistent with the typical “Western diet”, which has been linked to an increase in proinflammatory mediator production and a reduction in the synthesis of anti-inflammatory and antioxidant mediators [9]. The resultant proinflammatory state is associated with higher pain intensity, lower pain thresholds, and the persistence of pain [6]. Similarly, a “Southern diet” pattern, high in fried foods, processed meats, added fats, refined sugar and sugar-sweetened beverages was recently associated with a 41% increase in the relative risk of reporting pain in a national sample in the USA [10].

Current meta-analyses provide evidence that healthful dietary patterns (such as Mediterranean, vegetarian, and vegan), and modified intake of specific nutrients, are beneficial in reducing chronic pain [11, 12]. Dietary interventions for the management of CMP have included specific foods, nutrients, antioxidants, and prebiotic supplementation [6, 11, 12]. Antioxidant and anti-inflammatory properties of foods, alone or as part of nutrient-rich dietary patterns such as the Mediterranean diet, may act to neutralise oxidative stress and chronic inflammation associated with persistent pain [13]. There is, however, limited evidence to make specific dietary recommendations for people with chronic pain [14].

While it has been proposed that a healthy diet assists in the reduction of chronic pain [11, 12], there are several barriers that need to be considered in chronic pain populations. Studies suggest that “comfort eating”, where food is used as a coping strategy in managing chronic pain, is common across the body mass index (BMI) spectrum [15]. Chronic pain patients have a higher energy, sugar, and fat intake, and greater reliance on convenience or fast foods [7, 16]. Altered dietary behaviours in association with pain may, in addition to contributing to excess weight gain [15], lead to increased BMI in those with chronic pain [16, 17], and be a factor in lower diet quality scores [18].

Risk factors for poorer diet quality and weight gain associated with chronic pain include functional limitations affecting mobility and food preparation, and stress-related overeating [15, 19]. Additionally, the capacity to promote lifestyle changes may be influenced by the negative psychological association between pain and mood [19]. Low mood may affect motivation and self-efficacy, making engaging in health behaviours and weight loss more difficult [15]. Chronic pain adversely impacts sleep and physical activity, thus promoting sedentary behaviours which may also contribute to excess weight gain [20]. Adverse side effects of analgesics may equally influence appetite, contributing to poorer diet quality [7].

Pain influences eating behaviour, and accordingly may influence the relationship between body composition and CMP [15, 17, 20]. However, although studies suggest that levels of pain are associated with an increased energy intake, dietary intake during chronic pain episodes remains poorly characterised [16, 19].

To address the current paucity of understanding about dietary patterns associated with concurrent pain episodes, the primary aim of the study is to assess the feasibility of conducting a study that characterises concurrent pain experience and diet quality, and dietary behaviours in people with CMP. Feasibility criteria will evaluate participant recruitment, enrolment, retention, compliance with data collection processes (to assess concurrent pain, mood, sleep, and dietary intake, plus activity patterns), and participants’ perceptions on the study, with the goal to inform the future delivery of a dietary intervention. Feasibility and exploratory outcomes (demographics, physical measures, health-related behaviours, dietary intake, pain experience and psychological measures) will be reported using descriptive statistics.

## Methods

### Design

**Fig. 1:**
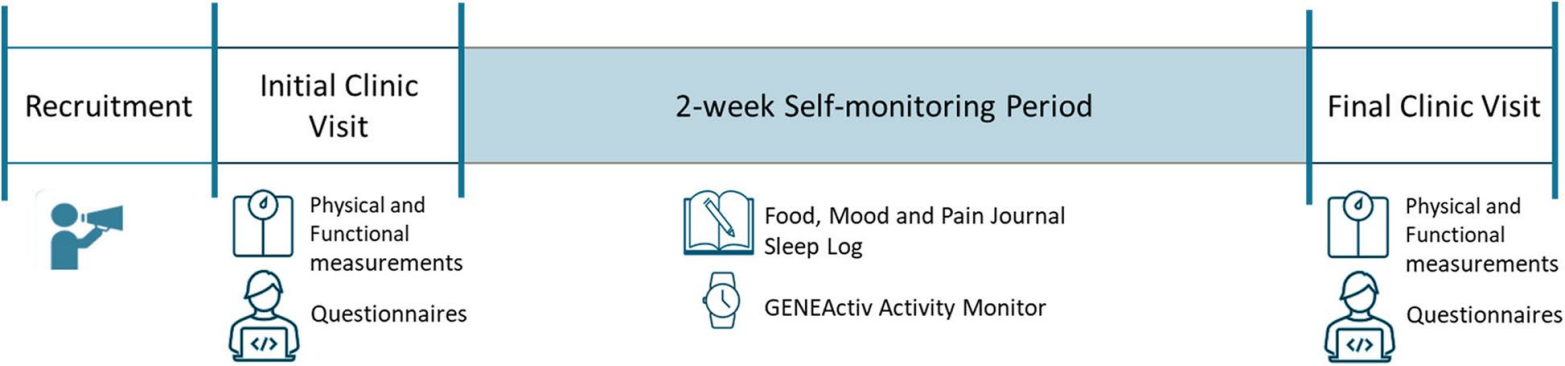
Study timeline

The study will be undertaken with individuals experiencing persistent musculoskeletal pain in a free-living environment. Participants will attend two in-person clinic visits where physical measurements and a series of pain and lifestyle questionnaires will be completed. Visits will be conducted pre and post a 2-week self-monitoring period where participants will wear a wrist-worn activity monitor (GENEActiv; for 2 weeks) and self-report concurrent diet, sleep, mood and pain on four randomly selected days (Fig. 1).

### Study setting and participant recruitment

This study will be conducted at the University of South Australia (UniSA) Clinical Trials Facility (CTF), Adelaide, South Australia, and The International Spine Centre (TISC), Adelaide, South Australia, an inter-disciplinary service for individuals with predominantly painful spinal conditions. Participants will be recruited from TISC and more broadly through the community within metropolitan Adelaide. Recruitment strategies will include advertisements placed on public notice boards and circulated via social media platforms, and online forums with a pain focus (such as Pain Australia, and Chronic Pain Australia). Interested individuals will be provided a Participant Information Sheet, and link to an online eligibility questionnaire and consent form on the UniSA Research Electronic Data Capture (REDCap) platform [21]. Consenting participants meeting eligibility criteria will be enrolled into the study.

#### Eligibility criteria

To be eligible for inclusion, participants must be aged 18 to 75 years who self-report CMP on 5 or more days of the week, for a period exceeding 3 months. Participants will be excluded if they are awaiting surgery for remediation of structural causes of their pain-provoking condition, have known or suspected serious pathological causes of pain (infection, fracture, cancer), neuropathic pain, headaches classified as migraine, cluster or tension type, orofacial pain, or fibromyalgia diagnosis. Individuals currently receiving dietary consultation, undertaking a weight loss programme, or having had previous bariatric surgery will be excluded. Participants should understand written and verbal English communication without the aid of an interpreter.

### Sample size

We aim to enrol 50 participants in 8 months. Drop-out rates reported in similar studies are ~ 30%, and sample sizes between 24 and 50 have been previously recommended for feasibility studies [16, 18, 22, 23]. Based on completion rates for a comparable study, the criteria for considering whether the concurrent pain, mood, and dietary intake data collection tool is feasible will be valid data collected on ≥ 75% of participants who complete the study [16]. As this feasibility study aims to evaluate compliance with data collection processes, these targets will allow for the completion of the full protocol in 26 participants.

### Data collection

A summary of the proposed data collection schedule, in accordance with the Standard Protocol Items: Recommendations for Interventional Trials (SPIRIT) guidelines, is outlined in Table 1.

**Table 1:** Summary of the proposed data collection schedule

	**Screening**	**Initial clinic assessment**	**2-week self-monitoring period**	**Final clinic assessment**
**Inclusion/exclusion criteria**	**X**			
Leeds Assessment of Neuropathic Symptoms and Signs (S-LANSS)	**X**			
**Informed consent**		**X**		
**Demographic data**		**X**		
**General health**		**X**		**X**
**Anthropometry**				
Height, weight, waist circumference		**X**		
**Functional capacity**				
Grip strength, timed up and go (TUG)		**X**		**X**
**Pain**				
Short form McGill Pain Questionnaire (SF-MPQ)		**X**		**X**
Body chart		**X**		**X**
Persistent Low Back Pain Questionnaire (PLBPQ) (back pain only)		**X**		
Visual analogue scales (VAS)		**X**	**X**	**X**
**Health-related quality of life**				
Short form-36 (SF36)		**X**		**X**
**Mood**				
Profile of Moods State (POMS)		**X**		**X**
Visual Analogue Mood Scales (VAMS)			**X**	
**Sleep**				
Pittsburgh Sleep Quality Index (PSQI)		**X**		**X**
Sleep log			**X**	
**Activity patterns**				
GENEActiv			**X**	
**Dietary intake**				
Weighed Food Record (WFR)			**X**	
**Diet quality**				
Dietary Guideline Index (DGI)			**X**	
**Physical activity**				
International Physical Activity Questionnaire (IPAQ)				**X**
**Pain catastrophising**				
Pain Catastrophising Scale (PCS)				**X**
**Dietary behaviours**				
Power of Food Scale (PFS)				**X**
Food Craving Scale (FCQ-State)				**X**
Control of Eating Questionnaire (Co-EQ)				**X**
Dutch Emotional Eating Behaviour Questionnaire (DEBQ-E)				**X**
Revised General Nutrition Knowledge Questionnaire for Australia (AUS-R NKQ)				**X**
**Food-related behaviours questionnaire**				**X**
**Exit survey**				**X**

#### Procedure

##### Initial assessment

Participants will attend an initial assessment at UniSA CTF or TISC. This visit will include obtaining written informed consent. Participant demographics, general health, diagnoses of pain-provoking condition(s), analgesia, and past injuries and/or surgeries will be documented. Questionnaires completed at this visit will capture participants’ pain experience, health-related quality of life, mood, and sleep. Anthropometric measurements and functional capacity assessments will be completed. Participants will be provided with a food diary, sleep log, and wrist-worn accelerometer (GENEActive, Activinsights, UK) to measure activity and sleep patterns during the 2-week self-monitoring period.

##### Two-week self-monitoring period

Participants will capture dietary intake on four non-consecutive days, across the 2-week period, using a weighed food record (WFR). At each eating occasion, participants will report their pain intensity (visual analogue scale (VAS)) and mood (Visual Analogue Mood Scales (VAMS)). Participants will complete a sleep log to report sleep times on the days preceding, and following, the day that dietary intake is recorded. The sleep log will contain a section to report wrist-worn accelerometer (GENEActiv) non-wear times. The accelerometer will be worn for the entire 2-week period.

##### Final assessment

A final assessment will be undertaken in the week following the 2-week self-monitoring period. Participants will complete questionnaires to capture dietary behaviours, physical activity levels, pain, and thoughts and feelings in response to pain. Participant experience and perspectives on the study will be captured with an exit survey.

### Feasibility outcomes and key feasibility criteria

Within the key metrics of feasibility [24], outcomes, specific to the process, resources, and scientific metrics of this study, are presented in Table 2.

**Table 2:** Feasibility outcomes and associated criteria

Feasibility metric	Feasibility measure	Key feasibility criteria
***Process:***		
Recruitment	Response to recruitment strategies	Proportion remaining interested in the study after provided study information and completing eligibility screening.
Refusal	Total number of eligible participants declining to participate	Proportion who met eligibility criteria but did not consent to participate.
Enrolment	Total number of participants enrolled	Enrol 50 participants in 8 months.
Retention	Total number of participants completing the protocol	Completed protocol in 35 participants (70%). Drop-out rates reported in similar studies are ~ 30%, and sample sizes between 24 and 50 have been previously recommended for feasibility studies [22, 23].
***Resources:***		
Compliance with data collection protocol	Weighed food record with concurrent pain and mood measures. Sleep log. GENEActiv wear time	Complete entries for 75% of participants who complete the protocol. A complete entry will include date and time of day of each eating occasion, food, and beverage intake (food and amount), with concurrent pain VAS and mood VAS rating, completed sleep log, and at least 4 days of ~ 20 h of GENEActiv wear time.
***Scientific:***		
Safety	Questionnaire	Absence of adverse effects.
Participant’s view on intervention	Exit survey	Proportion of participants reporting that self-reported data collection processes were burdensome. Proportion of participants satisfied with study setting and procedures (responding neutral and above).
Research identified as appropriate for the target group	Food-related behaviours questionnaire	Participant responses identify pain influences diet (proportion reporting agreement with statements).

### Outcome measures

**Fig. 2:**
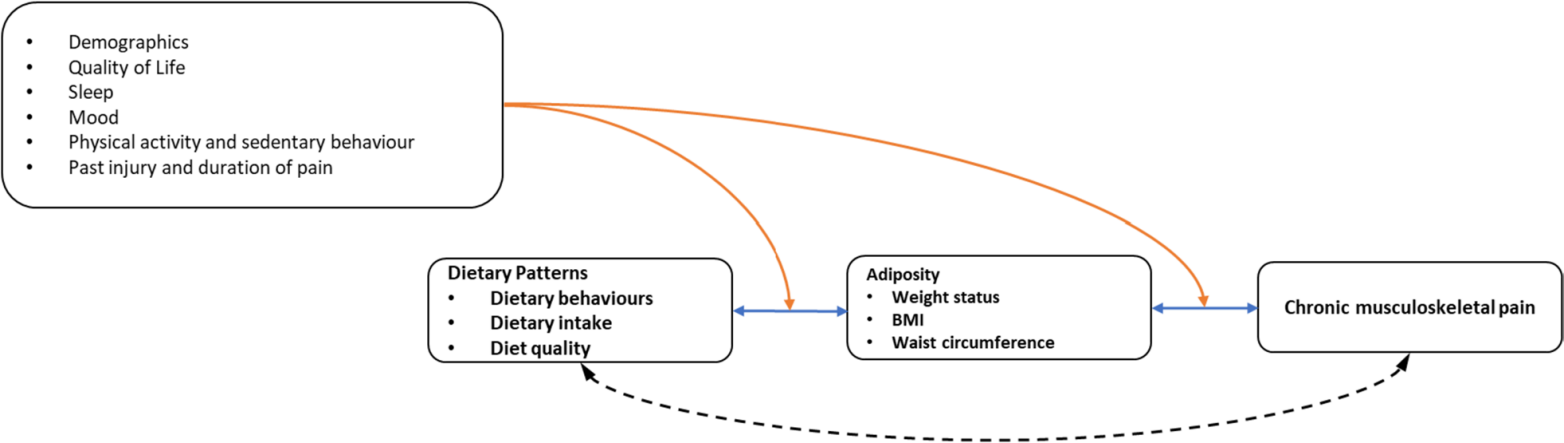
Conceptual model showing established bi-directional relationships between dietary patterns and adiposity, as well as adiposity and chronic musculoskeletal pain (blue lines). The extent to which dietary patterns (independent of weight status) are associated with CMP are not as well established and are the focus of the study, indicated by the dashed black line. Our analysis includes demographics and lifestyle behaviours that could induce confounding of associations, indicated with orange lines

Exploration of participant demographics and exploratory (non-feasibility) outcome measures have been included based on their suitability for use in a future dietary intervention in a CMP population (Table 3). These include demographic, physical, health-related, and psychological assessments related to the conceptual associations shown in Fig. 2.

**Table 3:** Summary of exploratory (non-feasibility) outcome measures

**Demographics**	Age, gender, life-stage (pregnant, breastfeeding, menopausal status), postcode, employment status, highest level of education, comorbidities, medication.
**Physical measures**	Anthropometry (weight, height, waist circumference); functional capacity (grip strength, TUG).
**Health-related behaviours**	Dietary intake (WFR); diet quality (DGI); nutrition knowledge (AUS-R NKQ); activity patterns (GENEActiv, IPAQ); sleep (sleep log, PSQI).
**Pain**	Neuropathic pain (S-LANSS); body chart (pain location and duration; diagnosis of pain-provoking condition, pain treatments, pain medication, past injuries/surgeries, compensable injury); persistent low back pain (PLBPQ); pain severity (SF-MPQ); pain intensity (VAS); pain catastrophising (PCS).
**Psychological measures**	Health-related quality of life (SF36); mood (POMS, VAMS); eating behaviours (hedonic hunger (PFS), present food craving (FCQ-S), food craving control (CoEQ), emotional eating (DEBQ-E)).

Abbreviations: *AUS-R NKQ* Revised General Nutrition Knowledge Questionnaire for Australia, *DGI* Dietary Guideline Index, *CoEQ* Control of Eating Questionnaire, *DEBQ-E* Dutch Emotional Eating Behaviour Questionnaire, *FCQ-S* Food Craving Scale, *IPAQ* International Physical Activity Questionnaire, *PCS* Pain Catastrophising Scale, *PFS* Power of Food Scale, *PLBPQ* Persistent Low Back Pain Questionnaire, *PSQI* Pittsburgh Sleep Quality Index, *POMS* Profile of Moods State, *SF36* Short-form 36, *SF-MPQ* Short Form McGill Pain Questionnaire, *S-LANSS* Leeds Assessment of Neuropathic Symptoms and Signs, *TUG* timed up and go, *VAMS* Visual Analogue Mood Scale, *VAS* visual analogue scale

#### Demographics

Participant information, as outlined in Table 3, will be captured via the REDCap questionnaire.

#### Physical measures

##### Anthropometry

Height (SECA 216 Height Measuring Rod, SECA) and weight (TANITA Ultimate Scale 2000, Tanita Corporation, Tokyo, Japan) will be measured in duplicate and averaged to calculate BMI (weight (kg)/height (m)^2^). Waist circumference will be measured to the nearest 1 mm using a metal measuring tape as per the International Society for the Advancement of Kinanthropometry protocol [25].

##### Functional capacity

Functional mobility will be assessed by the timed up and go (TUG) test. Participants are timed getting up from a seated position, walking 3 m, turning, returning to the chair, and sitting down again [26]. The TUG is conducted 4 times, the first practice trial is discarded, and the average time (seconds) of trials 2–4 is recorded. The coefficient of variation error for the TUG test in adults has been reported as 6% in previous studies [27].

Grip strength will be measured according to the Southampton protocol guidelines with a Jamar hand-held dynamometer (Lafayette Instrument Company, USA) [28]. Three repeat maximal attempts for right- and left-hand grip strength will be recorded with the maximal grip score from all trials compared to healthy age- and gender-matched individuals. Hand dominance and any reasons for testing exclusion will be documented. The validity of grip strength as a test of muscular function in nutritional evaluation has been demonstrated [29].

#### Health-related behaviours

##### Dietary intake and diet quality

In the 2 weeks following the initial assessment, participants will record dietary intake (including supplement and medication use) on four non-consecutive days, including three weekdays and one weekend day, using a specifically designed WFR. The four-day time frame was selected to balance the high participant burden associated with this method with the level of detail required to characterise usual dietary intake [30]. Participants will be instructed on which days/date to capture their dietary intake and to record times of food and drink consumption for each eating occasion on these days. The WFR will include a 100 mm long VAS to capture the participant’s concurrent pain intensity at each eating/drinking occasion. Daily VAS scores will be averaged to rate daily pain intensity. Six word-anchored 100mm VAMS will similarly capture concurrent mood at each eating/drinking occasion and will be averaged to rate daily mood.

Participants will be instructed to measure and record quantities of all foods and beverages consumed with as much detail as possible, including reporting names of branded products. Kitchen scales will be provided to participants who do not have access to their own. They will be guided to use standard household measures (tablespoon, cup, etc.) to estimate quantities when unable to weigh foods/drinks, such as when dining out. Dietary data will be checked for completeness and entered into FoodWorks Professional Nutritional Analysis Software, v10.0 (Xyris, Brisbane) to provide an estimate of total energy, micro- and macronutrient intake. Diet quality will be determined using the Dietary Guideline Index for application to WFR, reflecting age- and sex-specific Australian dietary guidelines [31].

##### Nutrition knowledge

Revised General Nutrition Knowledge Questionnaire for Australia (AUS-R NKQ) provides a consistent, reliable, and valid (Cronbach’s alpha = 0.92) measure of nutrition knowledge and current dietary recommendations in Australian adults. This is a 38-item questionnaire that assesses knowledge on dietary recommendations, nutrients in food, food choices and diet-disease relationships. Scores for each section and an overall score will be computed [32].

##### Activity patterns

Activity patterns (sleep, physical activity, time sedentary) will be measured with triaxial accelerometers (GENEActiv Original, Activinsights). Optimal behavioural variability in activity patterns can be collected via GENEActiv accelerometer data across 6 days, including weekend days [33]. Participants will wear the monitor on the wrist of their non-dominant hand 24 h/day for the 2-week self-reporting period, removing only for bathing/water-based activities. Activity and sleep data will be processed by GENEActiv software (GENEActiv PC Software, Activinsights), with non-wear time identified using the method of Choi et al. [34]. Data will be considered valid if it includes at least 4 days of approximately 20 h of wear time [35]. The sleep log will document times the device is removed (non-wear), put back on, and reason for removal (e.g. showering). At least 5 days of wear time has been recommended for reliable assessment of sleep patterns [36]. GENEActiv displayed very good sensitivity and accuracy (86% and 76% respectively) in inter-device comparisons of sleep monitoring (compared to Actiwatch-2, Phillips Respironics). Specificity was relatively low (40%), but values were similar to previous validation studies of actigraphs with polysomnography [37, 38].

Sleep logs are widely used in sleep research as a subjective measure of sleep [39]. A sleep log will record sleep (‘lights out’) and wake times (‘out of bed’) on the days prior to, during and post recording of food intake. Total sleep time and number of sleep bouts will be reported. Participants will also rate the quality of their sleep compared to a ‘normal’ sleep period. Sleep quality will be rated for each sleeping occasion as (1) very good, (2) good, (3) average, (4) poor, (5) very poor, or (6) did not sleep.

Sleep quality and disturbances over the previous month will be assessed using The Pittsburgh Sleep Quality Index (PSQI). The PSQI is a widely used reliable (alpha = 0.83) and validated measure of sleep quality [39]. Seven component scores reflect subjective assessments of sleep with component scores summed to give a ‘global’ score ranging from 0 to 21. Participants will be dichotomized into “good sleepers” (scores ≤ 5) and “poor sleepers” (scores > 5) [40, 41].

The International Physical Activity Questionnaire (IPAQ) will assess physical activity and sedentary behaviour at the final assessment [42]. The duration (minutes) and frequency (days) of physical activity in the last 7 days are measured across multiple domains; transportation (driving, walking, cycling), recreation (including sport and leisure time), housework, job-related, and time spent sitting. The IPAQ has been shown to be as reliable (Spearman’s *ρ* = 0.81) [42] and valid as other self-report methods of physical activity when compared against accelerometer measure of physical activity (*ρ* = 0.33) [43].

#### Pain

Pain assessment tools were selected from a variety of standardised tools designed to measure type of pain, pain severity and intensity, and functional impact of pain [44].

The self-reported Leeds Assessment of Neuropathic Symptoms & Signs (S-LANSS) will be used within the eligibility questionnaire to identify individuals with pain of neuropathic origin, who will be excluded from the study. The S-LANSS pain scale is a validated assessment tool, with scores of 12 or more out of 24 suggesting contributory neuropathic mechanisms [45].

Sites of pain experienced will be identified, and severity ranked, by participants on a body chart. Pain duration, diagnoses of pain-provoking condition(s), analgesia, past injuries and/or surgeries, and if the pain condition is from a compensable injury will be documented. A Persistent Low Back Pain Questionnaire (PLBPQ) will be employed to capture a minimum data set for participants who identify low back pain as their primary site of pain.

The Short Form McGill Pain Questionnaire (SF-MPQ) will provide a multi-dimensional measure of the nature of pain experienced at each site through 11 sensory and 4 affective words rated on an intensity scale. The SF-MPQ is suitable for the evaluation of pain complaints and to measure the effects of interventions or pain relief in individuals [46, 47].

Pain VAS (100mm) will rate the intensity of pain from 0 being ‘no pain’ to 10 ‘worst pain’. Pain VAS are highly acceptable and widely used subjective measures of pain intensity. The VAS test–retest reliability has been reported to be high (*r* = 0.94, *p* < 0.001) among literate patients [48]. A lack of a gold standard prevents criterion validity to be determined, but the VAS is highly correlated with verbal descriptions of pain; ranging from 0.62 to 0.91 [49] and is sensitive to treatment effects [50]. Participants will rate the intensity of their current pain for each pain site using a VAS at clinic visits. Pain VAS will also be incorporated in the food diaries to rate concurrent pain intensity during an eating occasion.

The Pain Catastrophising Scale (PCS) used to evaluate thoughts and feelings in response to pain, will be captured at the final assessment [51]. The PCS consists of 13 items used to generate a total score (0–53). Additionally, three subscales assess rumination, magnification, and helplessness. The PCS is widely used to measure catastrophic thinking in response to pain. It has demonstrated excellent internal consistency. The coefficient alpha for total PCS is 0.87–0.93, rumination (0.85–0.91), magnification (0.66–0.75) and helplessness (0.78–0.87) [51, 52].

#### Psychological measures

##### Health-related quality of life

The RAND Short-form 36 (SF36) questionnaire will be used for assessing health-related quality of life (HRQoL) [53]. It comprises of 36 items evaluating four physical health domains (physical functioning, role limitations due to physical functioning, pain, and general health) and mental health domains (energy/fatigue, social functioning, role limitation due to emotional problems and mental health). Scores for the SF36 domains range from 0–100, with higher scores reflecting a better HRQoL. Scores derived from the domains are aggregated into a physical component scale (PCS), and mental component scale (MCS), which will be used to evaluate general mental health status [54, 55]. The SF36 bodily pain subscales include two items that assess the intensity of pain and how pain interferes with usual occupation. It is widely used and has demonstrated good validity and reliability, with internal consistency (Cronbach’s alpha) 0.59 to 0.91 across four studies [47].

##### Mood

The Profile of Mood State (POMS) questionnaire measures six mood dimensions: anxiety, depression, irritability, vigour, fatigue and confusion [56]. POMS is a standard validated test with internal consistency Cronbach alpha from 0.63 (confusion) to 0.96 (depression). Total Mood Disturbance scores range from -32 to 200 with a cut-off score of 33 used to identify participants with significant mood disturbances [57].

Visual Analogue Mood Scales are a reliable and valid measure of mood states, with high reproducibility and validity in appetite assessment [58]. They offer a quick method to assess the current affective state, reducing the effect of reactance and fatigue [59, 60]. Using six word-anchored 100 mm VAMS, participants will indicate their happiness, sadness, calmness, tension, energy, and sleepiness on each eating occasion. Polak et al. [61] reported that measuring < 10 moods is appropriate for real-time approaches in daily diary formats for nutrition science, offering the opportunity to capture momentary changes in mood.

##### Eating behaviours

Food and eating-related behaviours will be captured with questionnaires administered at the final clinic assessment.

The Dutch Eating Behaviour Questionnaire emotional eating subscale (DEBQ-E) assesses eating behaviours in response to negative emotional states [62]. The DEBQ-E contains 13 items describing eating in response to diffuse (4), and clearly labelled emotions (9), and has shown high internal consistency and dimensional stability [62].

The Power of Food Scale (PFS) evaluates appetite motivation in the absence of energy requirements [63], assessing feelings of food control, through appetite-related feelings, thoughts, and motivations, in environments where palatable foods are consistently available [64]. The PFS has adequate internal consistency (Cronbach’s alpha 0.91) and test–retest reliability (*r* = 0.77, *p* < 0.001) [64]. Andreeva et al. [63] found a weak positive relationship between PFS scores and the brief Patient Health Questionnaire assessing anxiety and depression, concluding hedonic hunger may be coupled to emotional distress.

The Food Craving Scale (FCQ-State) [65] measures present moment food craving including positive and negative reinforcement from eating, anticipated lack-of-control overeating and physiological triggers [66]. Test–retest reliability over time for the FCQ-S is low (*r* < 0.60), as expected, as it is a dynamic state food craving measure [65, 66].

The Control of Eating Questionnaire (Co-EQ) assesses craving control, encompassing intensity and frequency of food cravings, difficulties in resisting eating and self-control of eating; craving for sweet and savoury, and positive mood [67]. The CoEQ is a reliable and valid measure in identifying behaviours and traits that predict intake, overeating, and measures of adiposity [67]. A strength of the Co-EQ measure is that it assesses specific food cravings and includes a positive mood subscale which facilitates the evaluation of associations between food cravings and mood [66]. Psychometric properties of the Co-EQ have been examined; Cronbach’s alpha values for the subscales were 0.88 for craving control, 0.74 for positive mood, 0.67 for craving for sweet and 0.66 for craving for savoury [67].

### Data collection methods

Research Electronic Data Capture (REDCap) secure web application managed by the University of South Australia will be utilised for data collection. Sleep, mood, and pain data captured in the self-monitoring period will be stored as hardcopies and transcribed to relevant analytical software. Weighed food records will be stored as hard copies and entered into FoodWorks (Xyris, Brisbane) for nutrient analysis. At the end of the study, all de-identified data will be exported to SPSS® Version 28.0 software (IBM Corp. Armonk, NY) for analysis.

### Data analysis

Data will be analysed descriptively to evaluate feasibility outcomes (recruitment, enrolment, retention, data completeness, safety, and acceptability of data collection procedures) using SPSS. The sample will be described using counts and proportions, means (standard deviation), and/or medians (Inter Quartile Range), depending on the distribution of the data. A CONSORT flow diagram will be used to illustrate participant flow through the study. Reasons for withdrawal will be recorded and included in the flow diagram. Secondary exploratory (non-feasibility) outcome measures will be analysed descriptively, and inferential statistics will be calculated but not quantified against criteria as this study is not a formal intervention [68].

### Ethical considerations

#### Ethics approval

Ethics approval for this study was granted by the Human Research Ethics Committee (HREC) of the University of South Australia (204076). Written informed consent will be obtained from participants at the initial clinic visit.

#### Adverse events

As this study is observational, and not a formal intervention, no potential risks have been identified. Participants will be advised to follow their habitual diet, and physical assessments will be non-invasive. Any adverse events will be noted and reported as per ethical requirements. These outcomes will form part of the feasibility criteria.

### Data management

Participants will be deidentified through assigning unique participant numbers. All data and information generated as part of the study will be kept confidential by the investigator, supervisors and individuals as listed on the ethics application, and will not be released to any party unless required by law. The investigator or other site personnel will not use this information and data for any purpose other than conducting the study. These restrictions do not apply to information that is necessary for disclosure in confidence to the HREC solely for the evaluation of the study, or information which is necessary to disclose to provide appropriate medical care to a study participant.

### Data ownership and dissemination

The study data and results will be owned by the University of South Australia. A de-identified summary of study findings will be provided to collaborators at TISC, and participants. Consenting participants will receive a copy of their individual anthropometric and dietary data. Results will be disseminated in a manuscript(s), and to relevant health professionals at conference presentations. All investigators will have the opportunity to be authors on manuscripts.

## Results

This study is currently in data collection. The results of this study, including feasibility and outcome measures are expected towards the end of 2023.

## Discussion

Current management of chronic pain includes self-management approaches where the individual takes an active role [69]. Patients seeking strategies for pain management are increasingly identifying dietary intake as a treatment priority [70], and guidelines endorsed by the European Pain Federation outline nutritional assessment as an important component in the management of chronic pain [71]. Despite this, and considering an increased recent interest in the relationship between dietary patterns and pain, dietary patterns are poorly defined in people experiencing CMP [6].

People with chronic pain experience a higher number of nutrition-related chronic health conditions with dietary intake a leading risk factor for morbidity [72], and a key driver of weight gain [70]. There is emerging evidence that dietary components may influence the pain experience, possibly through inflammatory mechanisms [19]. Furthermore, the role of pain-driving dietary intake, particularly during a pain episode, requires further investigation.

### Strengths

Strengths of this study are that it will advise on how feasible it is to recruit and retain participants with CMP, and which outcomes are appropriate to include in a future dietary intervention trial. Elucidating daily interactions between pain episodes and food intake within a free-living environment will provide novel data that may assist in defining dietary patterns and drivers of dietary intake in people experiencing CMP. Furthermore, the data collection tools will expand our current understanding of the relationship between lifestyle factors including eating behaviours, weight status, activity and sleep patterns and CMP.

### Limitations

A potential challenge to this study will be the retention and adherence of participants. Chronic pain alters motivation, impedes participation in activities of daily living, and may have a negative impact on mood [15]. These factors are likely to influence the retention and adherence of participants, which is expected to be low, similar to previous studies [16]. In addition, the intentional or unintentional modification to their usual diet is a persistent potential limitation of studies where participants are required to record their usual food intake. However, patients at a pain management service have identified nutrition and physical activity-related goals as treatment priorities [70], potentially motivating adherence. Reporting on the feasibility aspects of the study will provide important information on barriers to nutrition and lifestyle assessment in persons with CMP and advise on trial design for a dietary intervention.

## Conclusions

There remains a lack of evidence behind dietary advice as an adjunct for effective pain management [14]. Studies that establish the contributions of lifestyle behaviours, including dietary intake and eating behaviours, to chronic musculoskeletal pain could be useful in developing strategies to remediate chronic pain and maximise functional capacity.

### Supplementary Information

**Additional file 1.**

## Data Availability

Data sharing is not applicable to this article as no datasets were generated or analysed during the current study.

## Abbreviations

AUS-R NKQ: Revised General Nutrition Knowledge Questionnaire for Australia
BMI: Body mass index
CMP: Chronic musculoskeletal pain
Co-EQ: Control of Eating Questionnaire
CTF: Clinical Trials Facility
DEBQ-E: Dutch Emotional Eating Behaviour Questionnaire
DGI: Dietary Guideline Index
FCQ-State: Food Craving Scale
HREC: Human Research Ethics Committee
HRQoL: Health-related quality of life
IPAQ: International Physical Activity Questionnaire
MCS: Mental Component Scale
PCS: Pain Catastrophising Scale
PCS: Physical Component Scale
PFS: Power of Food Scale
PLBPQ: Persistent Low Back Pain Questionnaire
POMS: Profile of Mood State
PSQI: Pittsburgh Sleep Quality Index
REDCap: Research Electronic Data Capture
SF36: Short-form 36
SF-MPQ: Short Form McGill Pain Questionnaire
S-LANSS: Leeds Assessment of Neuropathic Symptoms & Signs
TISC: The International Spine Centre
TUG: Timed up and go test
UniSA: University of South Australia
VAMS: Visual analogue mood scales
VAS: Visual analogue scale
WFR: Weighed food record
YLDs: Years lived with a disability
