# Supplementary material for: Characterising concurrent pain experience and dietary patterns in people with chronic musculoskeletal pain: a feasibility study protocol
Source: Pilot Feasibility Stud. 2024 Jan 22;10:13. doi: 10.1186/s40814-023-01438-4 (PMC10801926; doi:10.1186/s40814-023-01438-4)
Supplement: Supplementary file 1 — Additional file 1. [file 40814_2023_1438_MOESM1_ESM.pdf]

University of South Australia

PARTICIPANT INFORMATION SHEET

Pain Experience and Lifestyle Behaviours Study.

To profile dietary intake and lifestyle behaviours in people with persistent pain.

Purpose of the Study

Lifestyle modification is an emerging approach for managing pain. Several nutrition related factors impact chronic pain, including weight status and dietary choices. However, there is currently insufficient evidence to advise on specific dietary recommendations for this population.

The aim of this study is to investigate pain-related dietary and lifestyle behaviours in people with chronic musculoskeletal pain to guide future interventions. This study will be conducted as part of a PhD program at the University of South Australia. The investigators responsible for the study are Susan Ward (PhD student), Dr Alison Hill, Prof Alison Coates, Assoc Prof Katherine Baldock and Dr Carolyn Berryman.

What it involves

The study involves two in-person visits that will be conducted before and after a 2-week self-monitoring period. These visits will be completed at the University of South Australia, Clinical Trials Facility. At the in-person visits you will complete a series of questionnaires, and some physical measures. During the 2-week monitoring period you will self-report your diet, sleep, mood, and pain on 4 days. The figure below summarises the study timeline.

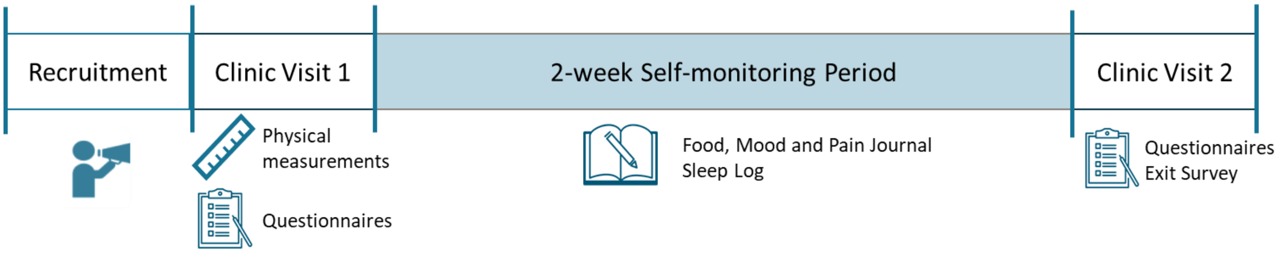

Figure 1. Study timeline.

Who is eligible for the study?

- To be eligible for the study you need to be:
  - Aged 18-75 years.
  - Experiencing chronic musculoskeletal pain on most days of the week for at least 3 months (pain arising from conditions of the muscles, bone, joints, or related soft tissues such as forms of arthritis, osteoporosis, and neck and back problems)
  - Willing to attend the clinic site for assessments
  - Able to understand written or verbal communication

- People who are not eligible for this study are those:
  - Awaiting surgery for remediation of structural causes of pain
  - With pain due to infection, fracture, or cancer
  - Headaches that are classified as migraines, cluster, or tension types, or orofacial pain
  - Neuropathic pain or fibromyalgia pain conditions
  - Receiving dietary consultations, undertaking a weight loss program, or having had bariatric surgery
  - Involved in any other study that may conflict with the study outcome measures
  - Are unwilling or unable to provide written consent

Study participation

If you would like to participate in this study, please contact **Louise Massie** (Clinical Trials Coordinator): **Email:** unisa.researchvolunteers@unisa.edu.au **Phone:** 8302 1365

The investigator will conduct a telephone screening to determine your initial suitability for the study. If you meet the criteria and are eligible, you will be invited to participate in the study, and we will schedule your first in-person visit at the Clinical Trials Facility (Bonython Jubilee Building, UniSA City East Campus, Frome Road).

You are welcome to discuss your participation in the study with your medical practitioner, or health professionals involved in your care, family, or friends.

Withdrawing from the study

Participation in the study is voluntary, and you may withdraw from the study at any time without prejudice. If you do choose to withdraw, you will be given the option at the time to decide whether you also withdraw consent to use your data that has already been collected. Withdrawal of your data will not be possible after completion of the final visit.

Obligations

You must be able to undertake the relevant assessment procedures during the study. You will need to inform the investigator of any changes to your health, medications, or ability to complete any requirement of the study, as changes to these may influence your ability to participate in the study or the trial.

In-Person Clinic Visit 1

The first clinic visit will take approximately 1.5 hours.

At this visit you will:

- Have the project explained to you in full by the study investigator. You will have the opportunity to ask questions before signing consent to be involved in the study.
- Confirm your general health, medical history, current treatments, and medication. This will include asking about various treatments you are undertaking including traditional or alternative therapies such as acupuncture and medications including over the counter medication, supplements, or herbal remedies.
- Have non-invasive physical measurements taken including height, weight, waist circumference, grip strength and mobility.
- Complete questionnaires about your quality of life, pain, and mood.
- Be provided a **Food, Mood & Pain Journal**
- Be provided a **Sleep Log**
- Be provided a wrist-worn **accelerometer**

2-Week Self-Monitoring Period

- During this 2-week period, you will be asked to record all food and drink intake on four days in your **Food, Mood & Pain Journal**. The research team will tell you which four days to record. At each eating or drinking occasion, you will also be asked to rate your mood and pain on 10cm line scales.
- You will be asked to complete a **Sleep Log** on the same four days you are reporting on your food, mood, and pain across the 2-week period.

You will be asked to wear the wrist-worn **accelerometer** at all times during the 2-week monitoring period. This device will record physical activity and sleep patterns during this period.

In-Person Clinic Visit 2

The second visit will take place at the end of the 2-week monitoring period and will take approximately 1 hour.

This visit will involve repeat physical measurements (grip strength and mobility), the completion of questionnaires, and a survey to report on your study experience. Your completed **Food, Mood & Pain Journal** and **Sleep Log** along with the wrist-worn **accelerometer** will be returned at this visit.

Description of Procedures

Height, weight, and waist circumference (10 minutes, initial visit only)

Height will be measured barefoot. Waist circumference will be measured with a tape at the midpoint of your torso on bare skin. Body weight will be measured barefoot and in light clothing on a scale. Your height and weight will be used to calculate Body Mass Index (BMI).

Grip Strength (5 minutes, initial visit only)

Will be measured by squeezing a handheld device (dynamometer) as hard as possible.

Functional Mobility (5 minutes, initial visit only)

You will be asked to sit on a chair and the time taken to stand from the chair, walk to a point across the room and return to a seated position in the chair will be timed.

Questionnaires (30 minutes, initial and final visits)

You will be asked to complete a series of questionnaires about quality of life, pain, and mood.

Food, Mood and Pain Journal (4 days entry)

You will be asked to measure and record all foods and drinks consumed on four specific days during this period. The Journal will include space to report the time of food/drink intake and your mood and level of pain at the time of food intake. You will be asked to record weights or estimated volumes of foods eaten using standard household measures, and record as much detail as possible about branded products. Kitchen scales will be provided if required.

Sleep Log (4 days entry)

You will be asked to record sleep times and naps on the same four days as the Food, Mood and Pain Journal.

Activity Patterns (approximately 5 minutes each day)

You will be asked to wear an activity monitor on your non-dominant wrist for 14 consecutive days during the self-monitoring period to measure your activity and sleep patterns. A log to record times the activity monitor is removed (e.g for showering) will be included within the Sleep Log.

Exit Survey (10 minutes, final visit only)

An exit survey will identify barriers to completing the study, and your satisfaction with the study as a participant.

Risks involved

All procedures will be carried out by trained and qualified personnel with strict occupational, health and safety guidelines. No specific risks have been identified beyond performing usual daily activities. The focus on pain and mood, however, may be distressing to you, and if needed, 24-

hour support resources can be found at Beyond Blue (www.beyondblue.org.au/ or phone 1300 22 4636) or Lifeline (www.lifeline.org.au/ or phone 13 11 14).

COVID-19

All visits will be compliant with current health directives with the following measures:

- All surfaces and equipment will be disinfected between sessions.
- Participants will be offered the option to wear a mask, and to ask the investigator to wear one during the session.
- Investigator will wash and sanitize hands.
- Physical distancing protocols will be maintained, and close contact will be minimised.

Privacy

On entering the study, you will be given an identification number (ID) which will be used for all data collection. Your name will not be used in any way for data entry or analysis or when results are published. Information and results from the study will be grouped for reporting, with individual responses not presented.

All records containing personal information will remain confidential and no information which could lead to identification of any individual will be released, unless required by law. The information collected in this study will be stored on a secure server at the Alliance for Research in Exercise, Nutrition and Activity's (ARENAs) secure data store in the Bonython Jubilee Building, City East Campus for a period of 15 years.

Benefits to study participation

You will not personally benefit from participating in the study, however you will be providing valuable data and contributing to the furthering of scientific knowledge. Participants who complete all aspects of the study will receive an honorarium of $40 to compensate for their time and travel expenses.

Within 6 months of completion of the study you will be provided with an analysis of your dietary intake and personal physical measures.

Costs

There are no costs involved in participating in this study.

Study funding

This study does not receive any external funding.

Contact

If you, or a member of your family, require more information about the study to help you arrive at a decision, please contact:

Clinical Trial Facility; Louise Massie: 8302 1365 **Email:** unisa.researchvolunteers@unisa.edu.au

Research Team

If you want any further information concerning this project or if you have any problems which may be related to your involvement in the project, you can contact the researcher, or any of the following people:

Name	Role	Phone	Email
Susan Ward	Researcher	83021365	susan.ward@mymail.unisa.edu.au
Dr Alison Hill	Supervisor	83021817	alison.hill@unisa.edu.au
Prof Alison Coates	Supervisor	83022313	alison.coates@unisa.edu.au
Assoc Prof Katherine Baldock	Supervisor	83022727	katherine.baldock@unisa.edu.au
Dr Carolyn Berryman	Supervisor	83131305	carolyn.berryman@unisa.edu.au

The ethical aspects of this research project have been approved by the Human Research Ethics Committee (HREC) of the University of South Australia as required by the Australian government research requirements, specified in the National Statement on Ethical Conduct in Human Research (2007 - updated 2018). Should you wish to discuss the project with someone not directly involved, in particular in relation to matters concerning policies, information about the conduct of the study, or your rights as a participant please contact the Executive Officer of UniSA HREC. Participants or third parties who wish to lodge a complaint about either the study or the way it is being conducted should contact the Executive Officer of UniSA HREC by email: humanethics@unisa.edu.au or telephone 83026330.

Project Consent Form

University of South Australia

Project Title: Pain Experience and Lifestyle Behaviours.

In signing this form, I confirm that:

1. I consent to take part in the research project entitled: **“Pain Experience and Lifestyle Behaviours”**.
2. I have read the Participant Information Sheet, and the nature and purpose of the project and the risks inherent in my participation have been explained to me. I understand and agree to take part.
3. Although I understand that the purpose of this research project is to understand food intake and lifestyle behaviours in a pain population, it has also been explained that my involvement may not be of any direct benefit to me.
4. I have been given the opportunity to have a member of my family or a friend present while the project was explained to me.
5. I have been informed that, while information gained during the study may be published, I will not be identified, and my personal results will not be divulged. Following the publication of results, data will be housed in a secure off-site storage facility run by the university and will be destroyed after 15 years.
6. I understand that I am free to withdraw from the project at any time and that this will not affect my rights or the responsibilities of the researchers in any respect.
7. I understand that if I withdraw from the study, I can choose at the time to withdraw consent for my data which has already been collected to be included.
8. I understand that my information will only be disclosed according to the consent provided, except where disclosure is required by law.
9. I understand that I will receive an honorarium of $40 upon completion of the study.
10. I am aware that I should retain a copy of this Consent Form, when completed, and the attached Participant Information Sheet.

Name of participant ………

Signed ……………………………………………………………………… **Date** …………………………………………………………

Witness

I have provided ……………………………………………………………………………… (Name of participant) information about the research and believe that he/she understands what is involved.

Researcher’s Name ………

Researcher’s signature …………………………………………………………….............. **Date** ....

Role in the project………

SAMPLE
